# Natural Product Polyamines That Inhibit Human Carbonic Anhydrases

**DOI:** 10.1155/2014/374079

**Published:** 2014-08-05

**Authors:** Rohan A. Davis, Daniela Vullo, Claudiu T. Supuran, Sally-Ann Poulsen

**Affiliations:** ^1^Eskitis Institute for Drug Discovery, Griffith University, Nathan, QLD 4111, Australia; ^2^Polo Scientifico, Laboratorio di Chimica Bioinorganica, Rm. 188, Università degli Studi di Firenze, Via della Lastruccia 3, Sesto Fiorentino, 50019 Florence, Italy

## Abstract

Natural product compound collections have proven an effective way to access chemical diversity and recent findings have identified phenolic, coumarin, and polyamine natural products as atypical chemotypes that inhibit carbonic anhydrases (CAs). CA enzymes are implicated as targets of variable drug therapeutic classes and the discovery of selective, drug-like CA inhibitors is essential. Just two natural product polyamines, spermine and spermidine, have until now been investigated as CA inhibitors. In this study, five more complex natural product polyamines **1–5**, derived from either marine sponge or fungi, were considered for inhibition of six different human CA isozymes of interest in therapeutic drug development. All compounds share a simple polyamine core fragment, either spermine or spermidine, yet display substantially different structure activity relationships for CA inhibition. Notably, polyamines **1–5** were submicromolar inhibitors of the cancer drug target CA IX, this is more potent than either spermine or spermidine.

## 1. Introduction

**Figure 1:**
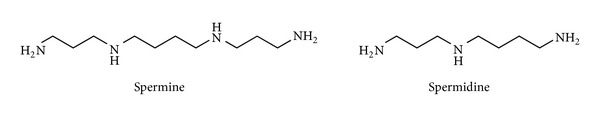
Natural product polyamine CA inhibitors, spermine and spermidine [10].

Carbonic anhydrases (CAs) catalyze the reversible hydration of carbon dioxide to bicarbonate anion and a proton: CO_2_ + H_2_O ⇆HCO_3_^−^ + H^+^ [1]. This equilibrium underpins a range of physiological processes including pH regulation, carbon metabolism, and ion transport. The therapeutic potential for modulating this reaction is well recognized across a number of diseases affecting humans, with the discovery that interfering with pH plays a major role in survival, growth, and metastasis of hypoxic tumours driving a need for small molecule CA inhibitors [2]. The active site of CA enzymes comprises a zinc cation that is coordinated to three conserved histidine residues and a hydroxide anion (OH^−^). The zinc-bound OH^−^ reacts with CO_2_ to generate HCO_3_^−^ and H^+^; these ions are then rapidly released to the microenvironment and the active enzyme is regenerated. The structural similarities in active site architecture across human CA isozymes are substantial and for drug discovery that is dependent on selectively targeting specific CA isozymes this presents a considerable hurdle [3]. Primary sulfonamide compounds (R–SO_2_NH_2_) coordinate as an anion (R–SO_2_NH^−^) to the CA active site zinc in place of the usual OH^−^ anion and are highly effective inhibitors of CAs. Many primary sulfonamide compounds are however nonselective, resulting in broad acting CA inhibitors that are a major drawback to drug discovery. The identification of new CA inhibitor chemotypes with better CA isozyme selectivity profiles is needed to address this drawback. Natural product (NP) compound collections have proven an effective way to access new chemotypes, and notably NPs have provided a significant portion of FDA approved drugs, particularly in the cancer therapeutics drug class [4]. Recent findings have identified phenol, [5–7] coumarin [8, 9], and polyamine [10] NPs that inhibit CAs. Using protein X-ray crystallography researchers have shown that each of these chemotypes interacts differently with the CA active site, and unlike primary sulfonamides none directly interact with the active site zinc [11]. The number of NPs that have so far been investigated for inhibition of CAs is however small and just a single innovative study that describes the inhibition of CAs with simple NP polyamines, spermine and spermidine, is reported (Figure 1) [10]. The inspiration for the present study is to further examine NP polyamines, particularly those with greater structural complexity than spermine and spermidine. With so few polyamines investigated for CA inhibitory activity, we hoped to broaden our understanding of the potential of polyamine alkaloids as an alternate nonclassical chemotype for CA inhibition.

Polyamines have been isolated from terrestrial and marine animals, plants, fungi, and bacteria [12]. These polycationic alkaloids are able to strongly interact with anionic biomolecules such as DNA and RNA and to a lesser extent proteins. This interaction may modulate a selection of cellular activities including gene expression, cell proliferation, translation, cell signaling, membrane stabilization, and ion channels [13–18]. The CA activity for two of the simplest NP polyamines, spermine and spermidine, and 16 semisynthetic polyamine analogues has been reported [10]. In this study, the inhibition of all 12 catalytically active human CA isozymes was assessed and the variation in* K*_*i*_ values ranged from low nanomolar to millimolar. The standout CA isozyme was CA IV, a transmembrane anchored enzyme with an extracellular orientated active site [19]. Both NP polyamines achieved lower* K*_*i*_ values, 0.010 *μ*M and 0.112 *μ*M for spermine and spermidine, respectively, at CA IV compared to all other tested CAs. An X-ray crystal structure of spermine in complex with CA II showed that it binds differently compared to primary sulfonamides. Spermine is a polycation at the experimental pH (pH = 7.4) and was found indirectly anchored to the zinc cation via the zinc-bound water ligand. Furthermore, a complex network of hydrogen bonds between spermine and CA active site residues was observed, including the terminal amine moiety which forms hydrogen bonds with residues Thr200 and Pro201. This CA binding mode suggests that NP polyamines may have potential to provide additional CA inhibitors with this altered mechanism of binding and inhibition of CAs. As polycations, compounds spermine and spermidine are not expected to enter cells by passive membrane diffusion, and this may give them some selectivity for extracellular facing CAs (CAs IV, IX, XII, and XIV) in cell models, and these isozymes are the focus of the present study.

## 2. Materials and Methods

### 2.1. Chemistry

Compounds were isolated from two marine sponge samples and one mushroom specimen, archived in the Nature Bank biota repository, [20] located at the Eskitis Institute for Drug Discovery, Griffith University. Nature Bank is a unique drug discovery resource that consists of >50,000 biota samples collected from Australia, China, and Papua New Guinea along with >200,000 fractionated natural product samples [21]. The five NP polyamines (**1**–**5**) investigated in this study were identified as previously reported NPs ianthelliformisamine A (**1**), ianthelliformisamine B (**2**), ianthelliformisamine C (**3**), spermatinamine (**4**), and pistillarin (**5**) following NMR and MS data analysis and comparison with literature values [17, 22–25]. Prior to biological evaluation all compounds were subjected to purity analysis by ^1^H NMR spectroscopy and shown to be >95%.

### 2.2. CA Inhibition Assay

An Applied Photophysics stopped-flow instrument was used for assaying the CA-catalyzed CO_2_ hydration activity [26]. IC_50_ values were obtained from dose response curves working at seven different concentrations of test compound; by fitting the curves using PRISM (http://www.graphpad.com/) and nonlinear least squares methods, values represent the mean of at least three different determinations as described by us previously [27]. The inhibition constants (*K*_*i*_) were then derived by using the Cheng-Prusoff equation [28] as follows:* K*_*i*_ = IC_50_/(1 + [S]/*K*_*m*_), where [S] represents the CO_2_ concentration at which the measurement was carried out and* K*_*m*_ is the concentration of substrate at which the enzyme activity is at half maximal. All enzymes used were recombinant, produced in* E. coli* as reported earlier [29, 30]. The concentrations of enzymes used in the assay were as follows: hCA I, 10.2 nM; hCA II, 9.5 nM; hCA IV, 8.9 nM; hCA IX, 8.7 nM; hCA XII, 10.9 nM; and hCA XIV, 12.6 nM.

## 3. Results and Discussion

**Figure 2:**
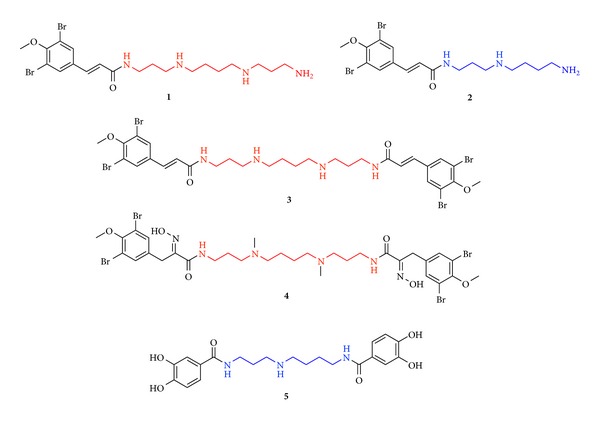
Natural product polyamines** 1**–**5 **sourced from the Davis compound library. Red: spermine core fragment and blue: spermidine core fragment.

The Davis Group at Eskitis has built up a unique in-house compound library over the past 10 years that currently consists of 352 distinct structures, the majority of which have been obtained from natural sources. Briefly, the NPs in this collection have been isolated from endophytic fungi, [31] macrofungi, [32] plants, [33], and marine invertebrates (e.g., sponges [34] and ascidians [35]) with quantities ranging from 0.4 mg to >1 g. Approximately 15% of this library contains semisynthetic NP analogues, [32, 36] while a small percentage (~5%) of the library is known commercial drugs or synthetic compounds inspired by NPs. A substructure search on this NP-based library against the spermidine fragment identified five secondary metabolites as hits. These included NP polyamines, ianthelliformisamines A–C (**1**–**3**), [17] spermatinamine (**4**) [22], and pistillarin (**5**) [23–25] (Figure 2), all of which have had various biological activities reported, but no CA bioactivity. Ianthelliformisamines A–C (**1**–**3**) were initially isolated from the marine sponge* Suberea ianthelliformis* and display varying levels of inhibitory activity against the Gram-negative bacteria* Pseudomonas aeruginosa* [17]. Specifically, ianthelliformisamine A (**1**) was the most potent antibacterial agent from this particular series with an IC_50_ value of 6.8 *μ*M (MIC 35 *μ*M) [17]. Spermatinamine (**4**) was originally isolated from the sponge* Pseudoceratina* sp. and was the first NP inhibitor of isoprenylcysteine carboxyl methyltransferase (ICMT), which catalyzes the carboxyl methylation of oncogenic proteins in the final step of a series of post-translational modifications [22]. ICMT has been proposed as an attractive and novel anticancer target [22]. More recently spermatinamine (**4**) and a series of related NPs have been shown to inhibit Gram-negative bacteria [18]. Pistillarin (**5**) has been isolated from a variety of fungal species including* Penicillium bilaii* [23],* Gomphus floccosus* [24],* Clavariadelphus pistillaris *[25], and several* Ramaria *species [25, 32] and is a known siderophore [37]. In addition, compound** 5** has recently been shown to exhibit significant protective effects against DNA damage caused by hydroxyl radicals generated from the Fenton reaction* via* iron chelation and to exhibit free radical-scavenging activity [24]. Pistillarin (**5**) and NP polyamines** 1**–**4** have all recently been evaluated for their antimalarial activity, [32] with** 4** and** 5** identified as the most potent polyamines towards a drug sensitive* Plasmodium falciparum* parasite line (3D7) with IC_50_ values of 0.23 and 1.9 *μ*M, respectively. These complex NP polyamines** 1**–**5** (Figure 2) form the basis of the present paper wherein we describe the CA inhibition against six human CA isozymes.

The inhibition activity data for the simple NP polyamines spermine and spermidine reported earlier and the more complex NP polyamines** 1**–**5** against six human CA isozymes of interest in therapeutic drug development is presented in Table 1. CA I and CA II are the predominant off-target isozymes (there are exceptions, e.g., CA inhibitors as antiglaucoma agents), while CAs IV, IX, XII, and XIV all have an extracellular oriented active site. CA IX and CA XII are overexpressed in many tumors [38, 39], CA IV plays a role in eye and kidney pathology [40, 41], while CA XIV is less well studied but has a role in a number of organs [42]. Polyamines** 1**,** 3**, and** 4 **comprise the polyamine fragment [–NH–(CH_2_)_3_–NR–(CH_2_)_4_–NR–(CH_2_)_3_–NH–] that is present in spermine (R = H), while polyamines** 2** and** 5** comprise the shorter polyamine chain [–NH–(CH_2_)_3_–NH–(CH_2_)_4_–NH–] of spermidine. Compounds** 1 **and** 2** are derivatized at one terminal amine group of the base polyamine fragment with the other terminal amine unmodified, while compounds** 3**–**5** are derivatized at both terminal amine groups and so lack a primary amine end group. Polyamines** 1**–**4** are metabolites derived from bromotyrosine, with compound** 4 **comprising even further structural complexity including two oxime groups and methylation of the two central amines resulting in tertiary amines in place of secondary amines within the central polyamine fragment. Polyamine** 5 **differs to compounds** 1**–**4** as it comprises two catechol end groups. As both catechol and oxime moieties feature in the structure of known metal ion chelators [43, 44] polyamines** 4 **and** 5 **have the potential for a companion action to any bioactivity based on CA enzyme inhibition.

The polyamines spermine and spermidine have two primary amine end groups and a flexible, linear polyamine backbone. In contrast, as described above, compounds** 1**–**5** are more complex in structure and these structural differences result in considerably altered SAR for** 1**–**5** compared to the simpler NP fragments (Table 1). There are a number of general SAR trends of** 1**–**5** that differ; these include (i) ~200-fold greater inhibition of off-target CA I and CA II than spermine, yet similar CA I and CA II inhibition to the shorter chain spermidine, (ii) inhibition of both the cancer associated CA isozymes CA IX and CA XII in the submicromolar range, which is better than both spermine and spermidine, and (iii) generally inhibit the other two extracellular CAs, CA IV and CA XIV, more weakly than both spermine and spermidine. Polyamine** 4** has greater structural complexity than all other polyamines of this study and has much weaker inhibition of CA IV and CA XII (>20 *μ*M), and this indicates that the greater complexity lessens the binding interaction with CA IV and CA XII. It is interesting that this lessened activity is not observed for the other four CAs, suggesting that the structural features of** 4 **may be interacting at CA active site hot spots [45]. Compounds** 1 **and** 2** are modified at only one terminal amine of the base polyamine fragment yet have very similar SAR to compounds** 3 **and** 5** that are modified at each end and lack a primary amine group. This SAR suggests that it may be the combination of both primary amine end groups of spermine and spermidine that underpin their differing CA enzyme inhibition profile. Future efforts from our groups will employ protein X-ray crystallography and molecular modelling to investigate if altered binding orientations of these NP polyamines do contribute to the differing SAR observed.

## 4. Conclusions

The discovery of new CA inhibitors with an alternate mechanism of inhibition to classical zinc binding functional groups can benefit from the unique chemical diversity provided in NP compound collections and so far is relatively underexplored. The structural features of polyamines** 1**–**5 **in this study, compared to the much simpler polyamine fragments of spermine and spermidine, further contribute to our understanding of the potential of both NPs and alternate chemotypes to contribute useful ligands to better enable the direction of the CA drug discovery field.

## Figures and Tables

**Table 1:** Inhibition data of human CA isozymes with simple natural product polyamines: spermine and spermidine [10] and complex natural product polyamines **1**–**5** and the clinically used CA inhibitor, acetazolamide.

Polyamine	*K*_*i*_ (*μ*M)^a^					
	CA I	CA II	CA IV	CA IX	CA XII	CA XIV
Spermine	231	84	0.010	13.3	27.6	0.86
Spermidine	1.40	1.11	0.112	1.37	44.1	1.00
**1**	1.76	0.41	6.72	0.20	2.81	2.12
**2**	0.77	0.37	9.10	0.35	3.48	2.28
**3**	0.86	0.35	9.08	0.27	3.50	6.96
**4**	0.85	0.48	>20	0.34	>20	2.72
**5**	0.79	0.34	7.03	0.36	4.21	1.52
Acetazolamide	0.25	0.012	0.074	0.025	0.006	0.041

^a^Errors in the range of ±5% of the reported value, from three determinations using a stopped-flow CO_2_ anhydrase assay.
